# Development and Characterization of Cellulose/Iron Acetate Nanofibers for Bone Tissue Engineering Applications

**DOI:** 10.3390/polym13081339

**Published:** 2021-04-20

**Authors:** Hamouda M. Mousa, Kamal Hany Hussein, Mostafa M. Sayed, Mohamed K. Abd El-Rahman, Heung-Myong Woo

**Affiliations:** 1 Department of Mechanical Engineering, Faculty of Engineering, South Valley University, Qena 83523, Egypt; 2 Institute for Veterinary Science, College of Veterinary Medicine, Seoul National University, Seoul 08826, Korea; kamaleldeen.youssif@vet.au.edu.eg; 3 Department of Animal Surgery, College of Veterinary Medicine, Assiut University, Assiut 71515, Egypt; 4 Mechanical Design and Materials Department, Faculty of Energy Engineering, Aswan University, Aswan 81542, Egypt; Mostafa_sayed91@energy.aswu.edu.eg; 5 Analytical Chemistry Department, Faculty of Pharmacy, Cairo University, Kasr-El Aini Street, Cairo 11562, Egypt; kabdelazim@gmwgroup.harvard.edu; 6 Department of Chemistry and Chemical Biology, Harvard University, 12 Oxford Street, Cambridge, MA 02138, USA; 7 Stem Cell Institute, College of Veterinary Medicine, Kangwon National University, Chuncheon 24341, Gangwon, Korea; woohm@kangwon.ac.kr

**Keywords:** cellulose acetate, iron acetate, iron ions, nanofibers mats, electrospinning, bone tissue engineering

## Abstract

In tissue engineering, design of biomaterial with a micro/nano structure is an essential step to mimic extracellular matrix (ECM) and to enhance biomineralization as well as cell biocompatibility. Composite polymeric nanofiber with iron particles/ions has an important role in biomineralization and collagen synthesis for bone tissue engineering. Herein, we report development of polymeric cellulose acetate (CA) nanofibers (17 wt.%) and traces of iron acetates salt (0.5 wt.%) within a polymeric solution to form electrospinning nanofibers mats with iron nanoparticles for bone tissue engineering applications. The resulting mats were characterized using field emission scanning electron microscopy (FESEM), transmission electron microscope (TEM), Fourier transform infrared (FTIR), thermal gravimetric analysis (TGA), differential scanning calorimetry (DSC), X-ray diffraction (XRD), and X-ray photoelectron spectroscopy (XPS). The resulted morphology indicated that the average diameter of CA decreased after addition of iron from (395 ± 30) to (266 ± 19) nm and had dense fiber distributions that match those of native ECM. Moreover, addition of iron acetate to CA solution resulted in mats that are thermally stable. The initial decomposition temperature was 300 °C of CA/Fe mat > 270 °C of pure CA. Furthermore, a superior apatite formation resulted in a biomineralization test after 3 days of immersion in stimulated environmental condition. In vitro cell culture experiments demonstrated that the CA/Fe mat was biocompatible to human fetal-osteoblast cells (hFOB) with the ability to support the cell attachment and proliferation. These findings suggest that doping traces of iron acetate has a promising role in composite mats designed for bone tissue engineering as simple and economically nanoscale materials. Furthermore, these biomaterials can be used in a potential future application such as drug delivery, cancer treatment, and antibacterial materials.

## 1. Introduction

Tissue engineering has a great potential in the biomedical field by constructing biocompatible materials that can interact with living tissues and organs [1]. Engineering mats that mimic native tissue features are considered as a promising strategy for replacing the damaged or degenerated tissues than the current conventional therapies that have impediments of morbidity of the donor-site and rejection of immune response [2,3]. Once engineered mats are implanted in the defect sites, they promote formation of tissue matrices and act as a mechanical support for the host tissues [4,5]. These developed materials should have adequate biocompatibility and structure similar to that of the native ECM in order to enable cells to grow and differentiate to specific tissues similar to their natural counterpart [6]. There is an increase intention to create highly porous patches/mats with properties like that of the natural bone for orthopedic tissue engineering [7]. These mats have properties that are close to tissues densities, thus polymers are widely used to produce customize implants [8,9]. Several polymers have been applied in bone tissue engineering applications such as polycarprolactone (PCL) [10], polyamide-6,6, chitosan [11], nylon 6 [12], and hybrid polymers such as (polyurethane/gelatin/nylon 6) [13]. Moreover, nanocomposite polymers including poly (Lactic Acid) and zinc oxide nanoparticles (ZnO NPs) [14] and PCL/Hydroxyapatite (HAp)/simvastatin electrospun nanofiber [15] can work as multifunctional materials for Mg-coated implants for bone tissue engineering. Our group have developed a composite nanofiber scaffolds with a three-layer structural design from different polymers as tri-layered nanofiber patches with stable mechanical and biocompatible properties [7]. Biocompatibility of polymers can be enhanced with organic/inorganic substitutes such as silk fibroin [16], hydroxyapatite (HAp) [15], ZnO [10], Fe_2_O_3_ [17] to improve the biomineralization process and thus biocompatibility. The early formation of Ca/P ions on the surface of polymeric scaffolds/mats is an indicator for apatite formation and therefore enhancement of bone tissue regeneration [18]. Cellulose acetate (CA) is an insoluble cellulose derivative that has been used as a biomaterial in various tissue engineering applications [19]. It can be easily fabricated onto nanofiber forms with adequate elastic modulus and tensile strength as well as a high degree of crystallinity [20]. For example, CA-based scaffolds/mats hold promising properties for mimicking the native ECM of bone tissue and ability to biomineralize in the physiological environment [21]. The 3D nanofiber mats from CA promote growth of osteoblasts under an in vitro environment and enhance the formation of immature bone in vivo [22,23].

Natural bone mainly consists of 69 to 80 wt.% from calcium and phosphate as inorganic minerals, in addition to proteins of 17 to 20 wt.% like collagen type 1 as an organic component and remining water content [24]. Collagen type I represents 90% of the proteins of the organic matrix. Despite the fact that iron is one of the trace elements in the inorganic matrix, it plays a role in bone health and homeostasis [25,26]. Additionally, during biochemical reactions of the basic cellular processes, iron has role in cell functions and metabolism [27,28]. Therefore, the amount of iron designed for bone tissue engineering is necessary. Iron oxide nanoparticles are ideal biomaterials owing to their biocompatibility and magnetic properties as well as their low toxicity. The developed tissue engineered scaffolds that contain iron could promote growth factors in the magnetic carrier and guide it to the iron site in the scaffold which can potentially improve tissue and bone repair [29,30]. More investigations and studies of iron oxide nanoparticles (IONPs) are still highly desired [31]. The particle size is an important factor towards cellular uptake and transport, and subsequently gene delivery. Moreover, particle size has an impact on the magnetic properties. For example, magnetic nanoparticles with size less than 20 nm showed superparamagnetic properties. In terms of biological assessment, the released iron ions showed enhancement in hypoxia-inducible factor-1α expression [32]. Due to its magnetic properties, iron (Fe) plays an important role in bone remodeling as inorganic materials in bone scaffolds. For example, Ismat Ullah et al. [33] synthesized a co-substitute of Sr^2+^/Fe^3+^ in HAp NPs for various biomedical applications such as bone grafting, hyperthermia-based cancer treatment, and drug delivery. The impact of the co-doped traces of Sr/Fe in HAp showed an osteoblastic proliferation and osteogenic differentiation towards MC3T3-E1 cells and excellent antibacterial activity [34]. Moreover, the co-substitute Sr/Fe within HAp has multifunctional properties, improving the mechanical hardness, blood compatibility, adhesion, and proliferation [35]. Zahra Pasandideh et al. [36] demonstrated that addition of Fe^3+^, Co^2+^, and dually doped nanoparticles showed high bioactivity by HAp formation in the surface of the synthesized materials.

Synthesis of ultrafine fibers mats for tissue engineering applications is widely produced using electrospinning [16]. Electrospun nanofibers have many advantages such as simple setup, high surface/volume ratio, and three-dimensional porous morphology close to the ECM of real tissues [37,38]. The main objective of the present work is to investigate and develop ionic co-substitution of iron ions/nanoparticles within CA nanofibers which can serve as useful biomaterials in terms of structure, chemical composition, biomineralization, and bioactivity properties for bone tissue engineering. An attempt here is devoted to developing nanocomposite mats form CA/iron nanoparticles with a simple and economic preparation method. The study hypothesizes that the presence of iron particles in CA mats can promote biomineralization and hence apatite formation of the mats. The role of iron particles in CA nanofibers was investigated with several characterizations such as XRD, XPS, TEM, thermal degradation, and biomineralization test. The presence of iron nanoparticles in CA-iron acetate mats was evaluated for cytocompatibility and evaluated using an in vitro MTT assay with a direct/indirect experiment. Cell attachment on the outer mat surface was evaluated via FESEM.

## 2. Experimental

### 2.1. Nanofibers Mats Fabrication

Cellulose acetate (CA) (average Mn ~30,000), Iron (II) acetate, Acetone, N, N-Dimethylacetamide (DMAc) solvent, and Hank’s balanced salt, (HBSS); from Sigma Aldrich, Egypt (Egyptian International Center for Import, Nasr City), were used. A 17 wt.% of CA solution was prepared in (Acetone: DMAC in 2:1) and CA/Fe mats were prepared by addition of 0.5 wt. % of iron acetate (0.017 mg) as trace optimized concentrations and stirring for 12 h. The solution was prepared at room temperature. Electrospinning was set up according to our previous work [7]. In short, an applied high DC voltage was set at ~19 kV and ~1 mA with a syringe (inner diameter = 0.52 mm) with pressurized pump and aluminum foil as a collector at a feed rate of 1 mL/h and 15 cm working distance between the two electrical sources.

### 2.2. Characterizations

The as developed mats were sputter coated with gold before FESEM image scanning (FESEM, QUANTA FEG 250, Thermo Scientific™ Quanta™, Hillsboro, OR, USA) to obtain high image resolution. Transmission electron microscope (TEM, JEOL 2100 PLUS, Peabody, MA, USA) was used to characterize the CA/Fe scaffold. The chemical interaction of CA and the CA/Fe mat was tested with Fourier transform infrared (FTIR) spectra in transmittance mode (Shimadzu FTIR-8400 S, Kyoto, Japan). Elemental composition was investigated with X-ray diffraction (XRD, Rigaku, Japan) at 5°/min and X-ray photoelectron spectroscopy (XPS, shimadzu Co., Kyoto, Japan). Thermal gravimetric analysis (TGA) and differential scanning calorimetry (DSC) (LINSEIS STA PT-1000, Robbinsville, NJ, USA) were performed to evaluate thermal degradation. The test was performed with 10 mg of each scaffold and heated to 600 °C at a rate of 10 °C/min. The mat’s thickness was assessed by a coating thickness gauge meter (Elcometer 456, Accuracy: ±(3% + 2 μm)).

### 2.3. Biomineralization Test

The biomineralization process of the developed mats under stimulation of bioenvironmental conditions was done using simulated blood fluid (SBF) at 37 °C. SBF preparation was detailed in the previous protocols [39,40]. The developed mats were soaked in 15 mL of SBF and incubated at 37 °C up to 15 days. Mats were then dried at the end of incubation time and morphology was observed using FESEM and biomineralization of formed apatite was analyzed via XRD.

### 2.4. Indirect Cytotoxicity Assay

Preparation of the preconditioned medium of CA and CA/Fe mats was performed after subjecting the sheets to sterilization using ethylene oxide gas followed by incubation in Ham’s F12 and Dulbecco’s modified Eagle’s medium (DMEM; Invitrogen, CA, USA) at a 1:1 ratio with 1% penicillin/streptomycin (p/s, Gibco, Grand Island, NY, USA). The incubation was set at 37 °C and 120 rpm for 72 h, with standard ratio of 0.2 g of scaffold/mL from culture medium, then centrifuged and filtered through a 0.4 μm membrane [41]. Human fetal osteoblastic cells (hFOB 1.19) were cultured in DMEM /Ham’s F12 containing 10% fetal bovine serum (FBS; Hyclone, Logan, UT, USA) with 2.5 mM L-glutamine (Gibco) and 1% p/s at 34 °C and 5% CO_2_ till reaching 70% confluence. Then, the cells were trypsinated and seeded at a concentration of 15 × 10^3^ for 24 h in 48-well plate. This was followed by removing of medium and addition of preconditioned medium (500 µL) with supplementation of 10% FBS. In the positive control, cells were cultured in mixture of medium with 20% dimethyl sulfoxide (DMSO) and considered as a cytotoxic control. The plates were incubated at different time intervals at 1, 3, and 7 days. The metabolic activity of cells was evaluated using a [3-(4,5-dimethylthiazol)-2-yl]-2,5-diphenyltetrazolium bromide (MTT) assay with 50 μL of MTT solution. A total of 5 mg /mL was added to the cell culture wells. After incubation at 37 °C for 4 h, the medium was aspirated and then 250 μL of DMSO was added to the wells to dissolve insoluble crystals of formazan. After complete solubilization of crystals, 100 μL was transferred from the well plates to 96-well plates for further absorbance measurements. The absorbance was estimated at 570 nm of wavelength using a spectrophotometer plate reader. The resulted absorbance of cell response was calculated as the percentage of cell activity that was exposed to the preconditioned medium compared to the negative control. For qualitative evaluation of cell viability, live/dead assay was done using (calcein-AM/ethidium Bromide homodimer, Invitrogen) following the manufacturer’s recommendations, then fluorescence microscope images were captured after 7 days (Olympus, Tokyo, Japan).

### 2.5. Mats Cell Attachment

CA and CA/Fe were placed in 48-well plates to assess biocompatibility and adhesion of hFOB 1.19 cells. In brief, a suspension of cells and medium containing 25 × 10^3^ cells in a total of 500 μL were seeded on the resized mats surface followed by incubation at 34 °C in 5% CO_2_. After 2 h of incubation, the rounded mats were placed in another plate, so the remaining attached cells were kept without disturbance, thereafter 16 h of incubation at 34 °C, the MTT assay was performed.

### 2.6. Cell Proliferation on the Nanofiber Mats

A suspension of cells (15 × 10^3^ of hFOB 1.19) was evenly distributed on the top surface of CA and CA/Fe rounded sheets with 8 mm diameter placed in cell culture of 48-well plates. The MTT assay was used to measure the cell proliferation after 1, 3, and 7 days of culture. Further studies were performed using FESEM to check the attachment of cells in the mat surface. The polymerase chain reaction (PCR) was performed for the seeded cells on the scaffold surfaces after isolation of the RNA compared to cells cultured as a monolayer using TRIzol reagent (Invitrogen) according to manufacturer’s instructions and our reported previous work [39]. The sequences of the primers used for PCR amplification are appended in Table S1.

## 3. Results and Discussions

### 3.1. Characterization of the Developed Mats

**Figure 1:**
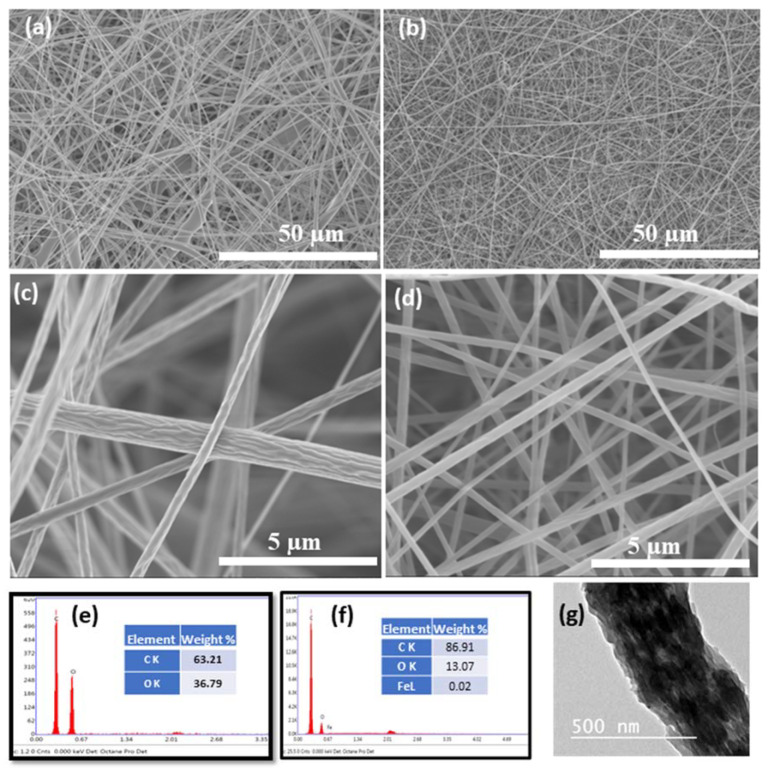
FESEM images of the different mats and EDS point analysis. (**a**,**c**) CA low and high resolution, and (**b**,**d**) CA/Fe mats. at low and high resolution (**e**,**f**) EDS point analysis of both CA and CA/Fe mats. (**g**) TEM image of the CA/Fe mats.

**Figure 2:**
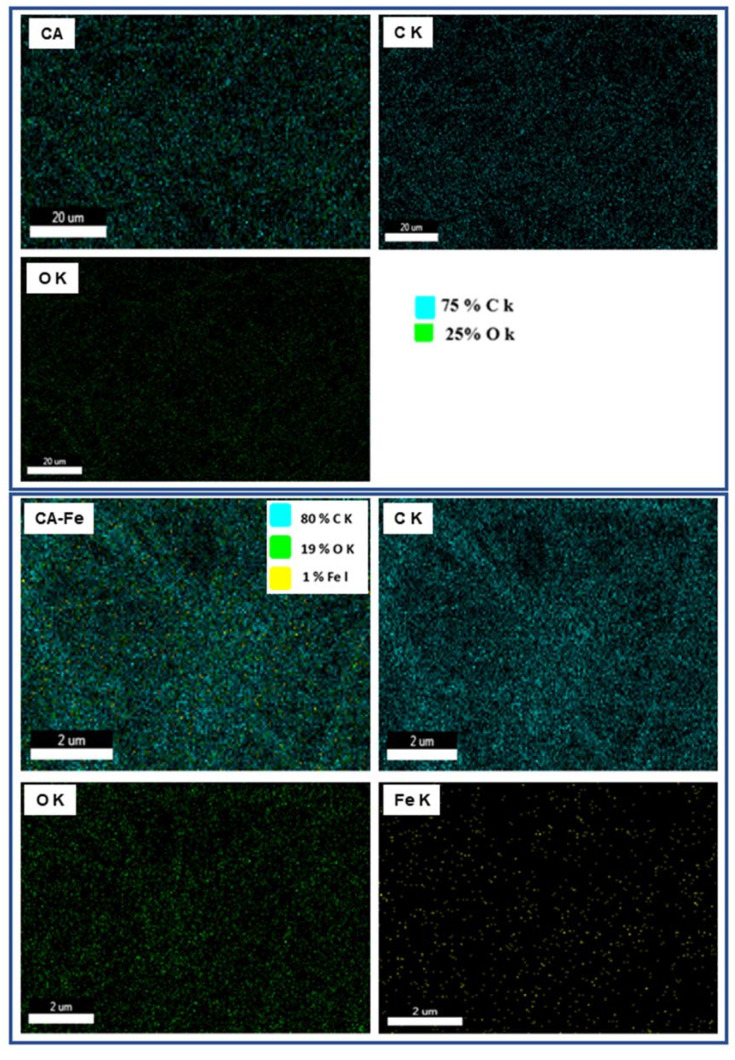
Figure illustrates the EDS mapping images of the two developed mats (labeled from left as CA and CA/Fe).

Nanofiber morphology was investigated with FESEM as shown in Figure 1a,b. The developed mats showed a smooth fiber distribution without any beads and randomly oriented as-spun nanofibers due to the application of the fixed collector. The presence of iron salt in electrospinning solution showed a dense fiber formation and subsequently fiber diameter was decreased with smooth surface morphology. However, compared to the neat CA scaffold, CA/Fe mat fiber diameter was obviously reduced, and dense fiber formation existed after incorporation of iron salt, as was clear from high resolution images in Figure 1c,d. The fiber diameters of the two mats were measured using ImageJ software and showed that both CA nanofiber and CA/Fe mats were 395 and 266 nm, respectively. These changes in morphology after addition of iron acetate to electrospinning solution facilitates fiber elongation during the electrospinning process and increasing of solution conductivity [42,43]. EDS analysis was conducted with FESEM to indicate the presence of iron in the resulting CA nanofiber. EDS results showed mainly formation of three elements which are C, O, and Fe in the fabricated mats as shown in Figure 1e,f. Element weight percent show traces of Fe in the CA/Fe mat with 0.02 wt.%. The CA/Fe mats were analyzed with TEM as shown in the inset image in Figure 1g, which shows that iron particles are distributed among the CA fiber without agglomeration and form a composite mat. In addition, EDS elemental mapping in Figure 2 shows the overall element distribution and individual elemental mapping. The CA mat shows 75% C and 25% O elements, which refers to the chemical composition of the polymer. In addition, the CA/Fe mat has a homogenous elemental distribution with elemental composition of 80% C, 19% O, and 1% Fe. The EDS analysis and elemental mapping clearly showed that the iron ions were uniformly distributed in the nanofiber and verified its presence at 1% of elemental percent. The developed CA and CA/Fe nanofiber were measured and had a 95 ± 6 and 99 ± 4 µm of thickness, respectively. The slightly increasing of the mats could attributed to the dense fiber formation comparing to the pure polymer.

### 3.2. Physiochemical Properties

**Figure 3:**
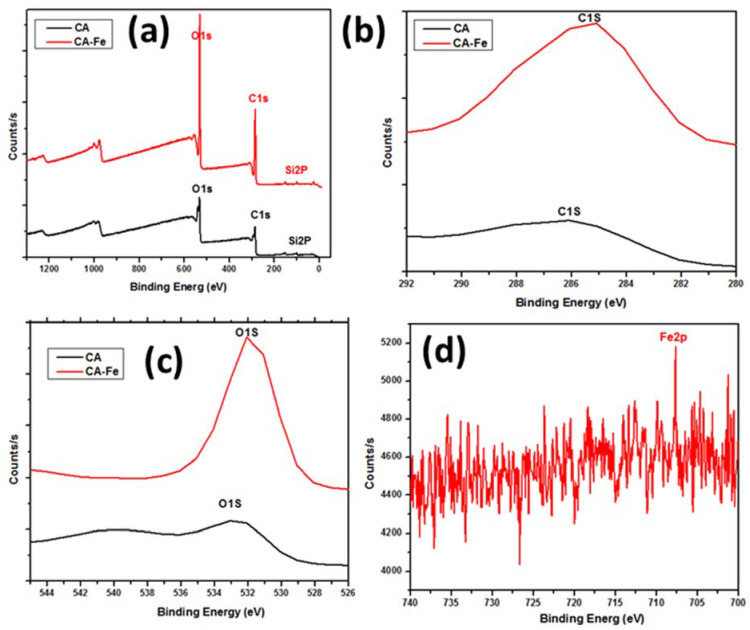
XPS analysis: (**a**) CA and CA/Fe mats wide scan analysis, (**b**) narrow carbon scan, (**c**) narrow oxygen scan, and (**d**) iron scan in CA/Fe mats.

**Figure 4:**
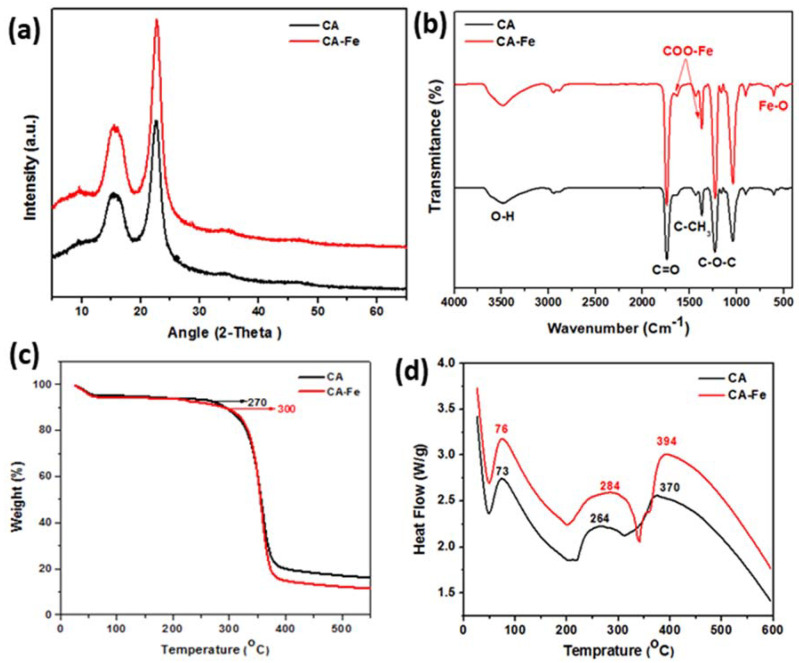
Mat characterizations for physiochemical and thermal properties: (**a**) XRD analysis and (**b**) FTIR analysis. (**c**) TGA analysis and (**d**) DSC analysis.

XPS results showed the elemental distribution on the outer surface of mats. Figure 3a shows XPS wide scan results of both CA and CA/Fe mats, the main peaks spectrum of C1s at 297.98 eV and O1s at 544.98 eV. The CA/Fe composite nanofiber mat has an Fe2p peak at 707 eV which demonstrates the iron distribution among the fibers [44]. The narrow scan for each element is presented in Figure 3b–d. Elementary compositions obtained from the survey spectrum are appended in Table 1. The survey spectrum of the samples revealed the presence of carbon with C = 63.32 at. %, oxygen with O = 36.68 at. %, and iron with Fe = 0.44 at. %. The crystal structure of the mats is shown in Figure 4a. It shows XRD patterns of both CA and CA/Fe composite nanofibers. As indicated, the characteristic peaks are shown at 2θ of 12.7° and 23.5°, corresponding to the crystallographic planes (101) and (002) of cellulose II diffraction [45]. The sharp peak indicates that the crystallinity of CA nanofibers is high [46]. Moreover, the CA/Fe mat indicates the presence of basic reflection of iron located at a 2-theta value of 30.1°, 35.5 °, and 43.2° attributed to planes of (220), (311), and (400), respectively [44,47,48].

Figure 4b shows FTIR spectrum of pure CA nanofiber and CA/Fe composite nanofiber. Pure CA nanofiber showed a characteristic absorption band of C=O stretching at 1752 and 1236 cm^−1^ attributed to acetate substituent presented by C–O–C alkoxyl stretching [49]. In addition, the band at 3480 cm^−1^ was attributed to O–H stretching vibration, and 1368 cm^−1^ was attributed to C–CH_3_ methyl bending [50]. The lower wave numbers (≤700 cm^−1^) are attributed to Fe–O vibration and hence attributed to the formed iron oxide nanoparticles [51]. The peak at 598 cm^−1^ in CA/Fe confirms the presence of the Fe–O bond [52]. In addition, absorptions at 1390 and 1588 cm^−1^ are characteristic peaks of the COO–Fe bond [53]. We can conclude that both CA and iron acetate are well blended and form nanocomposite fiber mats under high DC potential of electrospinning.

Thermogravimetric analysis (TGA) and differential scanning calorimetry (DSC) were carried out to obtain the thermal stability and degradation profiles of pure CA nanofiber and CA/Fe composite nanofiber. The TGA and DSC curves were presented in Figure 4c,d. The two scaffolds exhibited quite close TGA profiles as indicated in Figure 4c. The weight loss of CA scaffolds in a temperature ranges from room temperature to 100 °C, which, owing to the water evaporation and the second temperature range being between 250 and 300 °C, was attributed to decomposition of the cellulose matrix. On one hand, TGA results showed that no weight loss occurred at 500 °C on the neat CA nanofiber. On the other hand, the composite mats do not show any influencing changes in the glass transition temperature T_g_ of the pure CA scaffolds. The T_g_ of both mats was observed at 60 ± 10 °C. The CA/Fe mat initial decomposition temperature is 300 °C, which is higher than that of the pure CA mat at 270 °C. Additional investigation of DSC results is shown in Figure 4d. DSC shows exothermal behavior for both pure CA nanofibers and CA/Fe composite nanofibers mats and depicted thermal and energy kinetics energy. The two mats displayed an endothermic peak at a temperature from 50 to 100 °C. The endothermic peak of CA is due to the outflow of water which agrees with the observed TGA thermogram. The results exhibited an exothermic peak at 264 °C for the pure CA nanofibers which is attributed to the decomposition of CA. Comparison of this thermogram with that for the CA/Fe composite nanofibers was performed, which exhibited an exothermic peak at 284 °C. Further, pure CA mats have a main peak at 370 °C and this peak reached 394 °C in case of the CA/Fe mats. The results confirm introducing Fe to the CA polymer leads to a slight shifting of the thermal decomposition of CA to a higher temperature.

### 3.3. Biomimetic Mineralization

**Figure 5:**
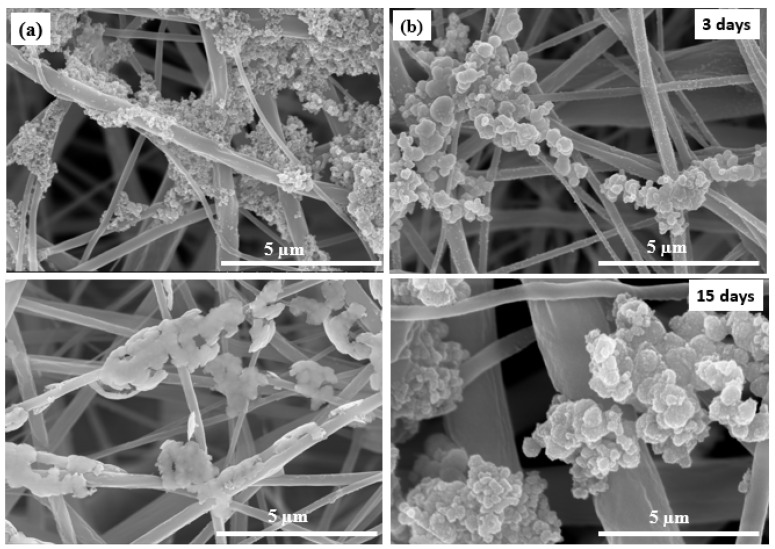
Biomineralization test shows FESEM images of mat morphology after SBF incubation for 3 and 15 days. (**a**) CA and (**b**) CA/Fe mats.

**Figure 6:**
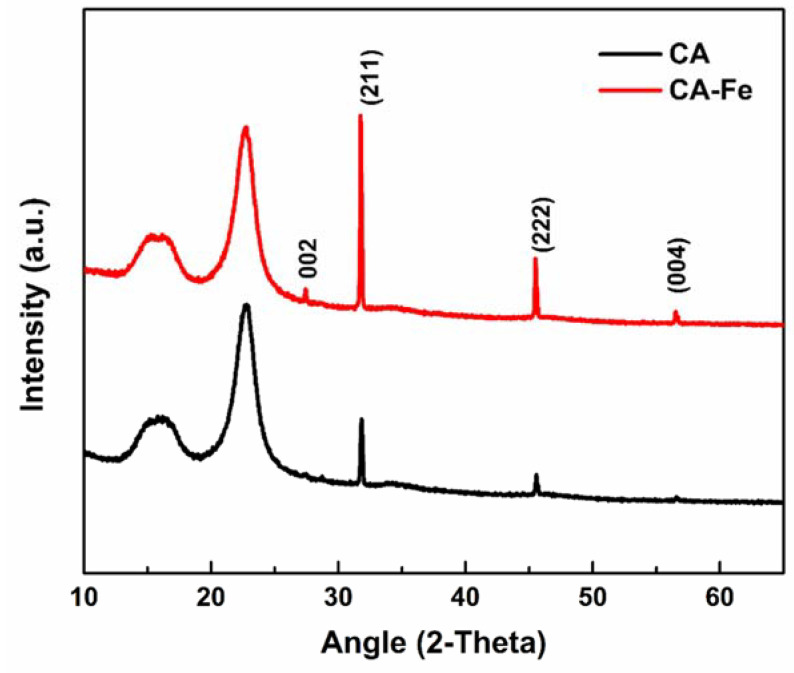
XRD of the different mats after 15 days of biomineralization.

The complex porous structure of bone has apatite crystals precipitated on the surface of a nanofiber and protein network [54]. Hence, biomimetic nucleation with apatite in spherical-like shapes on the mat surface is a real proof of mats/scaffold’s bioactivity and ability to bond with living natural bone [55]. Mats were evaluated in a physiological medium at a similar biological environment. The first assessment of the biomineralization is shown in Figure 5, which shows mat surface morphology after immersing in SBF medium at 37 °C up to 15 days. During biomineralization of mats, the exchange of ions in the mats outer surface and SBF solution is assigned to the apatite formation mechanism. The mechanism is based on the surrounding medium pH value and then formation of an apatite layer rich with Ca/P. This allows enhancement of the apatite ionic activity in the medium and produces a clear surface with a low energy interface conducive to the nucleation of apatite on the surface of nanofibers [56]. In addition, by increasing the immersion time, the formation of apatite particles was monotonically increased. Herein, apatite particles were more nucleated after 15 days of immersing in SBF. The CA/Fe mat showed more increasing in apatite particle formation than on the pristine CA mats at the same interval. The second assessment is XRD patterns that were performed for the two mat after a 15-day immersion in the SBF solution to prove the formation of apatite. Figure 6 shows XRD analysis of the two scaffolds, and there is a clear peak at 31.8° and 46.5° corresponding to (211) and (222) planes, which meet the standard data of apatite (Card: JCPDS No. 09-0432) [57]. In addition, minor peaks 2-theta of 27.5°, 57°, and 66.5° are attributed to apatite crystal structure plane of (002), (222), and (004), respectively [58]. The XRD results demonstrated the apatite particle formation on the pristine CA nanofibers. Interestingly, the intensity of peaks was sharp after iron addition. These peaks of CA/Fe after immersion have higher crystal structure owing to the formation of apatite particles higher levels of crystallinity. The apatite particles formed in CA/Fe composite nanofiber mat have a higher crystal structure which agrees with FESEM images.

### 3.4. Biocompatibility

**Figure 7:**
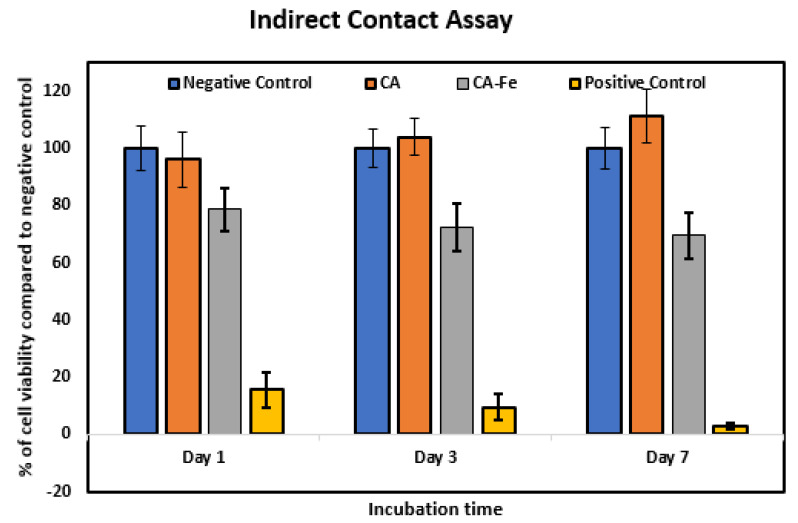
Cytotoxicity evaluation by indirect contact assay. The effect of the extracts of CA and CA/Fe on human fetal osteoblasts cultured for 1, 3, and 7 days measured by the MTT assay. Results are presented as means ± standard deviation (n = 8).

Biocompatibility of implanted materials is an important property that is responsible for host tissue response. In vitro, biomaterials can be evaluated using cytotoxicity tests [7]. Figure 7 shows the results of the indirect cell culture test and viability of seeded cells on the different mats. Results showed that there was a non-significant level of cell growth and proliferation between hFOB cultured using non-conditioned medium (negative control) and those cultured using preconditioned extracts from CA after 1, 3, and 7 days of culture. Cells cultured using extracts from CA/Fe displayed a lower viability and proliferation in comparison to the negative control. This decrease can be attributed to the higher concentration of iron ions in the extraction medium prepared from CA/Fe and used for culture of cells for 1, 3, and 7 days. In a biological system, iron is the most important element that can act as an electron donor [15,16]. However, more accumulation of iron intracellularly may induce the more production of reactive oxidative stress (ROS) and therefore cause cell injury. There are limited studies that evaluate bone cell cytotoxicity when cultured in the presence of iron ions that may be caused by induced ROS production leading to disruption of cell membrane integrity [59]. This suggests that iron-doped nanofiber can work as an antibacterial effect, which is in agreement with the previous reported work [34].

**Figure 8:**
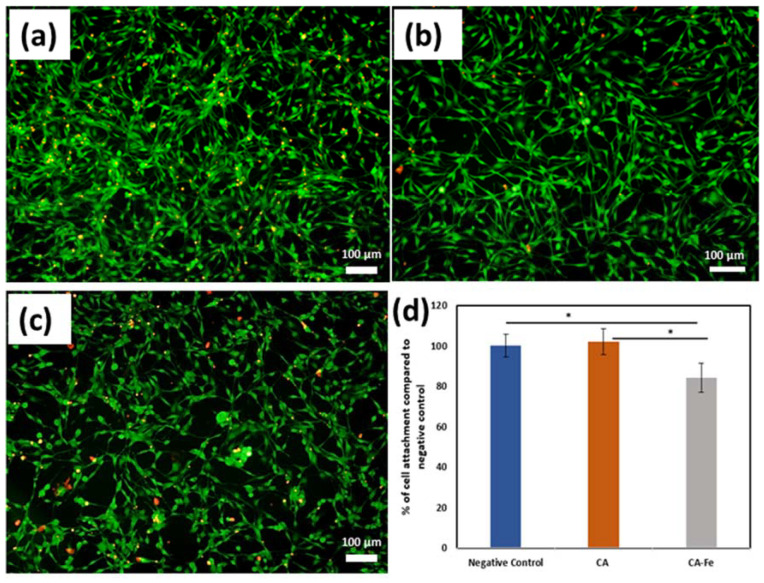
Fluorescent images of hFOB cells cultured as (**a**) negative control, or in presence of preconditioned medium of (**b**) CA, or (**c**) CA/Fe mats for 7 days. Calcein AM (green) indicates live cells while ethidium homodimer (red) indicates dead cells (scale bar =100 µm, magnification = 10×. (**d**) Cell attachment assay on CA and CA/Fe mats. A non-significant level of hFOB cell attachment is observed between CA mats and negative controls. Results are represented as means ± SD, * *p* ≤ 0.05; n = 8; Student’s *t* test.

**Figure 9:**
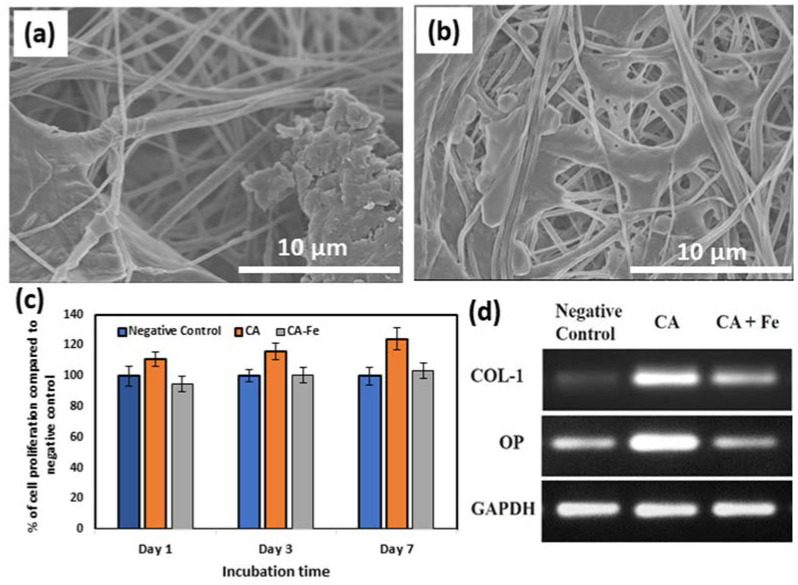
FESEM images of cell attachment: (**a**) CA and (**b**) CA/Fe mats. (**c**) Proliferation of hFOB cells grown on the top surface of CA and CA/Fe mats for 1, 3, and 7 days. Results are presented as means ± SD. (**d**) RT-PCR of the osteogenic related genes electrophoresed in 1.5% agarose gel.

Figure 8a–c shows preconditioned medium prepared using CA mats confirmed a low level of positive EthD-1 staining signals after 7 days of incubation. This proves that CA mats have no leachable toxic chemicals. However, more cells exhibited a positive behavior to EthD-1 staining upon exposure to preconditioned medium prepared from CA/Fe that is consistent with the results of the MTT assay. Cell adherence to the mat surface was evaluated using an attachment assay. In MTT assay, there was a significantly higher percentage of attachment for hFOB cells seeded on CA (102.15 ± 6.38) than CA/Fe sheets (84.23 ± 7.24) compared to the negative control cells as shown in Figure 8d. Moreover, Figure 9a,b shows cell attachment on the outer surface of the developed mats using FESEM images. The resulting images confirm that CA/Fe mats that contain iron promote cell growth and proliferation. Mats that contain iron particles have the highest levels of cell attachment and compared to CA mats, more cell proliferation is obtained. Further study performed using direct seeding of hFOB cells on disks of CA and CA/Fe mats showed they have a non-significant cell proliferation when comparing their results with the negative control after the first day of incubation, as shown in Figure 9c. A higher cell proliferation was clear at 3 and 7 days for CA mats with a 116.1 ± 5.6% and 123.9 ± 7.13% content, respectively. The expression of osteogenesis-related genes including Col-1, and osteopontin (OP) was quantified by PCR analysis for hFOB cells seeded on CA and CA/Fe mats. As shown in Figure 9d, the expressions of Col I and OP were significantly higher in cells grown on the CA mat than both cells grown on CA/Fe and cells cultured as a monolayer in a culture dish.

**Figure 10:**
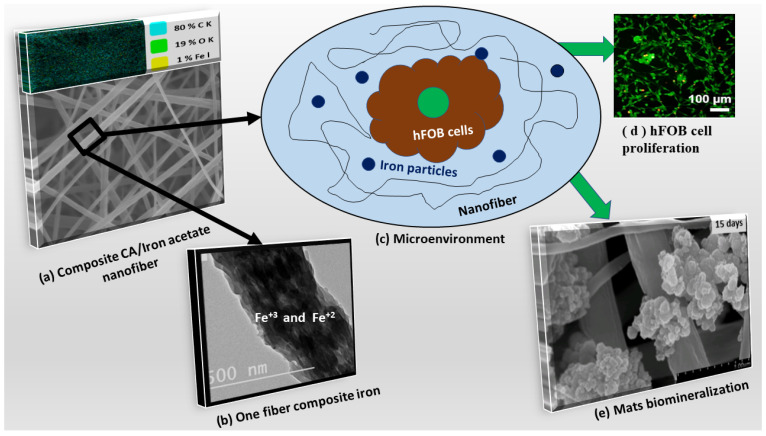
Scheme of composite mats and the microenvironment: (**a**) mat morphology with elements map distribution, (**b**) TEM image of fiber and iron nanoparticles distribution, (**c**) microenvironment and interaction with nanofiber mats, (**d**) hFOB cell proliferation, and (**e**) apatite formation after 15 days of incubation in a similar microenvironment.

The presented bioactive molecules in the implanted biomaterials mats influence biomineralization and gene regulation [60]. In the present work, Figure 10 shows a scheme of the composite CA/iron mats and the surrounding microenvironment. On one hand, the results demonstrated the biomineralization process successfully form apatite rich in Ca/P ions [18] which was proven in the biomineralization section in the present study in both FESEM and XRD analysis. The osteoblast cell line (hFOB) showed adequate proliferation using an in vitro test. The co-precipitation of iron oxide NPs with CA nanofiber showed enhancement of cell proliferation and attachment as indicated in Figure 9a,b and Figure 10d. The static magnetic field of such nanoparticles generates a mechanical force which in turn inhibits toll-like receptor activation and subsequently enhances growth factors expressions. Scaffolds/ mats containing magnetic nanoparticles have positive influences in different cell types, in specific osteoblast [61] and endothelial cell lines [62]. The enhancement of osteogenesis of osteoblasts of the superparamagnetic scaffold was achieved upon magnetization which is in agreement with the biomineralization result in Figure 10e [61]. Shengfa Zhu et al. [63] studied endothelial cell metabolic activity after exposure to different iron concentrations. Their finding showed that metabolic activity can be enhanced at iron ions less than 10 μg/mL and low metabolic activity at iron ions (>50 μg/mL). In addition, Mo-Tao Zhu et al. [64] explained that iron oxide nanoparticles induced endothelial system inflammation in three ways, which are escaping nanoparticles from phagocytosis and then interacting with the endothelial monolayer, then influencing the endothelial cells as free ions after dissolving of nanoparticles, and finally oxidative stress responses, as shown in Figure 10c. Another study by Ismat Ullah et al. [34] showed that the co-doped nanofiber with traces of Fe^3+^ ions have antibacterial activity against *S. aureus* and *E. coli* bacteria, and this is due to the interaction of irons ions with oxygen and forming of a complex. Overall, the proposed composite nanofiber opens the venous system for several applications such as multimodal biomedical imaging, catalysis, cancer therapy, targeted drug delivery, and diagnostics as a simple and economic nanomaterial.

## 4. Conclusions and Future Prospective

In conclusion, we successfully developed a nanocomposite mats by electrospinning from cellulose acetate and traces from iron acetate for bone tissue engineering applications. Results showed that addition of iron acetate to the electrospinning solution enriched it and resulted in decreasing in diameter compared to pure CA nanofiber. The addition of iron acetate could notably enhance the thermal stability of the pure CA mats. In addition, the presence of iron acetate was revealed to highly contribute to apatite particle formation, illustrating enhanced bioactive behavior of the CA/Fe composite nanofibers, and suggesting iron composite with CA can facilitate formation of apatite-like mineral deposition on the mat surface. Furthermore, in vitro cell culture seeded with hFOB cells have no cytotoxicity effect and could enhance and promote osteoblast cell attachment and proliferation among mats’ porous structure. The presented nanostructure is close to natural ECM with excellent biocompatibility. Future work can be extending by studying magnetic properties, antibacterial properties, mechanical and dielectric properties of the different mats, and future applications including drug delivery and cancer treatment.

## Figures and Tables

**Table 1:** XPS data analysis of different peaks from the different developed mats.

Mat Name	Name	Start BE	Peak BE	End BE	Height CPS	FWHM eV	Area (P) CPS.eV	Area (N) KE^0.6	at. %
CA	C1s	297.98	285.83	279.18	5405.41	5.44	29,575.44	419.99	63.32
	O1s	544.98	532.36	525.18	10,351.88	3.28	43,725.31	243.25	36.68
CA/Fe	C1s	297.98	285.30	279.18	10,839.70	4.76	46,563.45	661.05	63.28
	O1s	544.98	531.93	525.18	22,166.45	2.73	68,147.68	379.01	36.28
	Fe2p	739.98	707.71	700.18	412.90	0.10	4137.15	4.64	0.44

## Data Availability

No new data were created in this study. Data sharing is not applicable to this article.

## Supplementary Materials

The following are available online at https://www.mdpi.com/article/10.3390/polym13081339/s1, Table S1: Primers used for PCR analysis.
