# Supplementary material for: Development and Characterization of Cellulose/Iron Acetate Nanofibers for Bone Tissue Engineering Applications
Source: Polymers (Basel). 2021 Apr 20;13(8):1339. doi: 10.3390/polym13081339 (PMC8072972; doi:10.3390/polym13081339)
Supplement: Supplementary file 1 [file polymers-13-01339-s001.zip › polymers-1170777-supplementary.pdf]

Supporting information for:

Development and Characterization of Cellulose /Iron Acetate Nanofibers for Bone Tissue Engineering Applications

Hamouda M. Mousa[1*], Kamal Hany Hussein[2,3], Mostafa M. Sayed[4], Mohamed K. Abd El-Rahman[5,6], Heung-Myong Woo[7]

[1]Department of Mechanical Engineering, Faculty of Engineering, South Valley University, Qena 83523, Egypt.

[2]Institute for Veterinary Science, College of Veterinary Medicine, Seoul National University, Seoul 08826, Republic of Korea.

[3] Department of Animal Surgery, College of Veterinary Medicine, Assiut University, Assiut 71515, Egypt.

[4] Mechanical Design and Materials Department, Faculty of Energy Engineering, Aswan University, Aswan 81542, Egypt.

[5] Analytical Chemistry Department, Faculty of Pharmacy, Cairo University, Kasr-El Aini Street, Cairo, Egypt 11562.

[6] Department of Chemistry and Chemical Biology, Harvard University, 12 Oxford Street, MA 02138, United States.

[7] Stem Cell institute, College of Veterinary Medicine & Institute of Veterinary Science, Kangwon National University, Chuncheon, Gangwon 24341, Republic of Korea.

***Corresponding author:**

H.M.Mousa (**hmousa@eng.svu.edu.eg**)

Table. S1 Primers used for PCR analysis.

Primer	Primer sequences		Annealing Temperature °C
	Forward	Reverse	
**Collagen I**	5’- CAG CCG CTT CAC CTA CAG C -3’	5’- TTT TGT ATT CAA TCA CTG TCT TGC C -3’	57.1
**Osteopontin**	5’- CTC AGG CCA GTT GCA GCC -3’	5’- CAA AAG CAA ATC ACT GCA ATT CTC -3’	-
**GAPDH**	5’-ACA GTC AGC CGC ATC TTC TT-3’	5’-GAC AAG CTT CCC GTT CTC AG-3’	59.7
